# Supplementary material for: Investigation and Determination of Kinetic Parameters of Sweeteners Based on Steviol Glycosides by Isoconversional Methods
Source: Foods. 2025 Mar 31;14(7):1233. doi: 10.3390/foods14071233 (PMC11988719; doi:10.3390/foods14071233)
Supplement: Supplementary file 1 [file foods-14-01233-s001.zip › foods-3505241-supplementary.pdf]

Investigation and Determination of Kinetic Parameters of Sweeteners based on Steviol Glycosides by Isoconversional Methods

Naienne da Silva Santana [1], Sergio Neves Monteiro [2], Tatiana Carestiato da Silva [3] and Michelle Gonçalves Mothé [1,*]

[1] Department of Organic Processes, School of Chemistry, Federal University of Rio de Janeiro,
Rio de Janeiro 21941909, Brazil; naienne.santana@gmail.com
[2] Department of Science and Technology, Military Engineering Institute, Rio de Janeiro 22290270, Brazil; snevesmonteiro@gmail.com
[3] National Institute of Industrial Property, Rio de Janeiro 20090910, Brazil; tatiana_carestiato@hotmail.com
* Correspondence: michelle@eq.ufrj.br

Supplementary Material

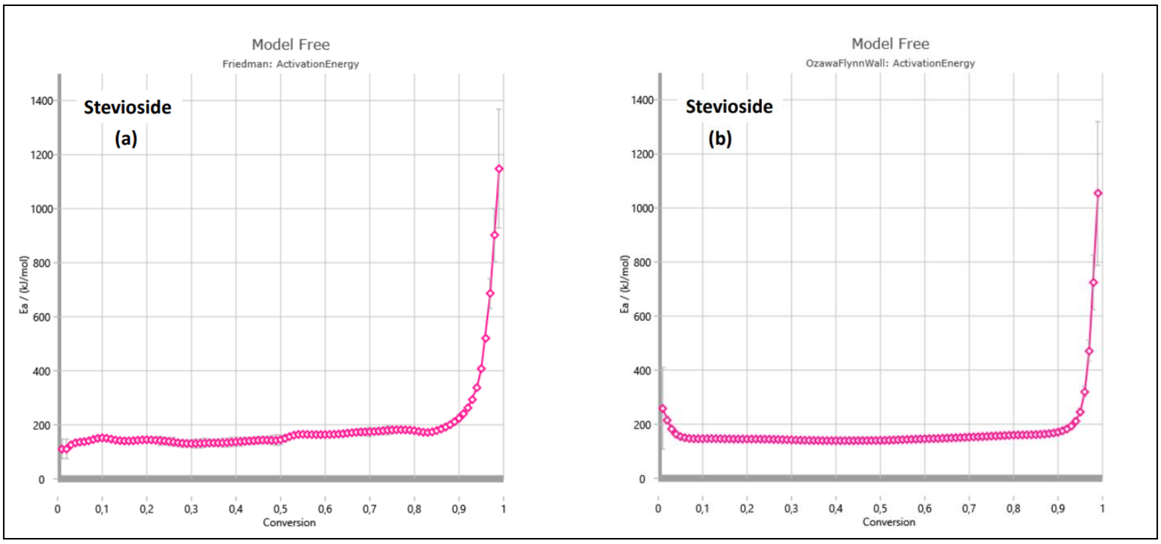

Figure S1. Graphic of E_a versus converted fraction (α) in (**a**) Friedman and (**b**) Ozawa-Fynn-Wall analysis for stevioside.

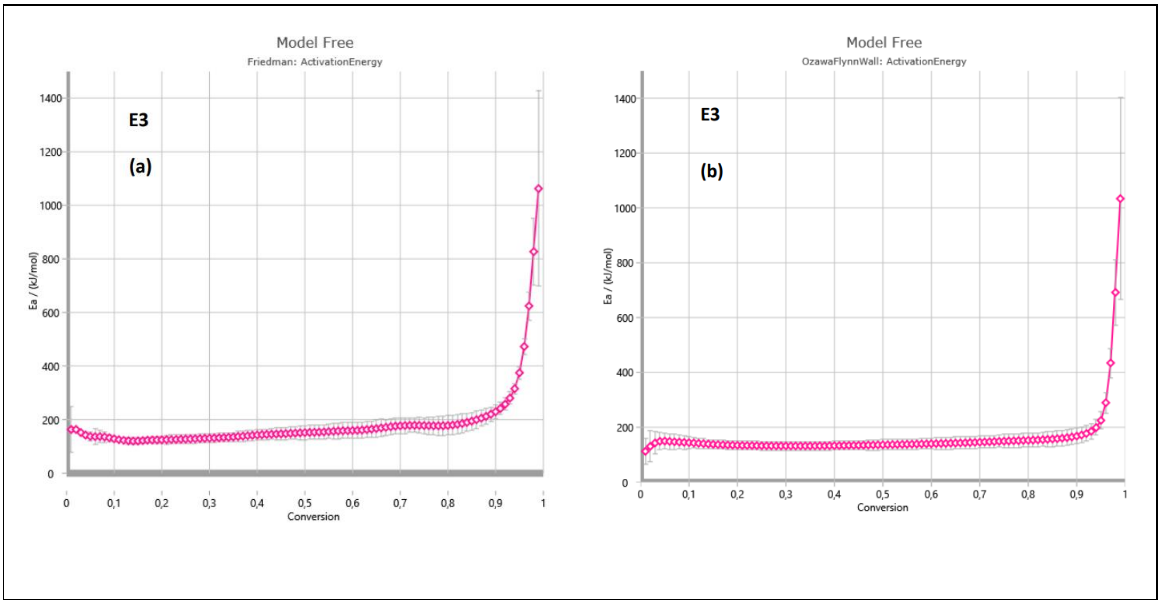

Figure S2. Graphic of E_a versus converted fraction (α) in (**a**) Friedman and (**b**) Ozawa-Fynn-Wall analysis for E3 sample.

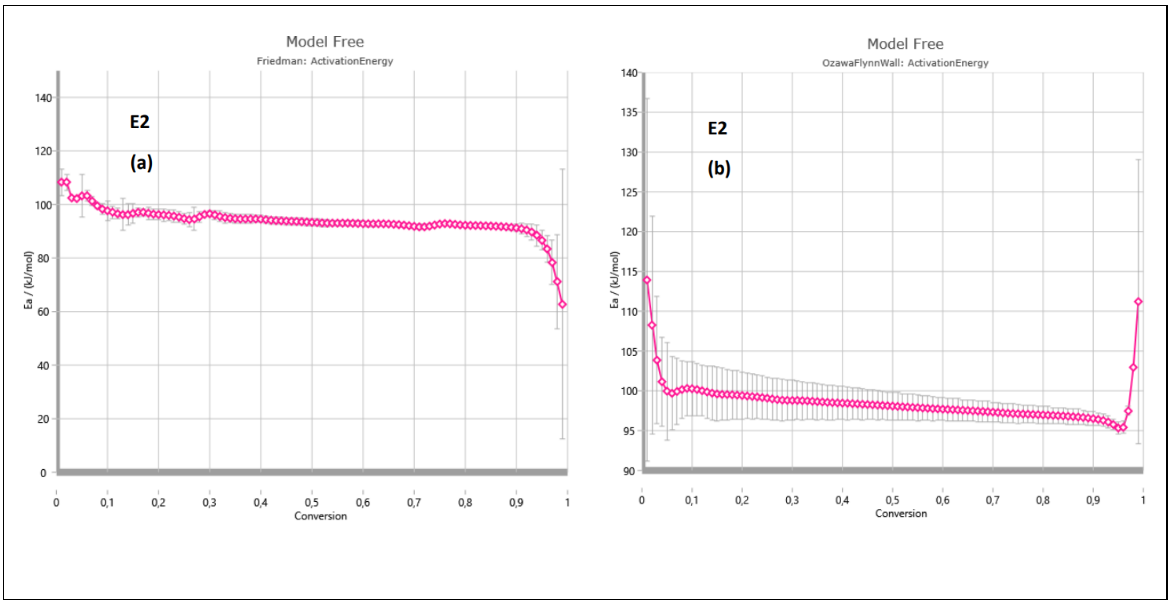

Figure S3. Graphic of E_a versus converted fraction (α) in (**a**) Friedman and (**b**) Ozawa-Fynn-Wall analysis for E2 sample.

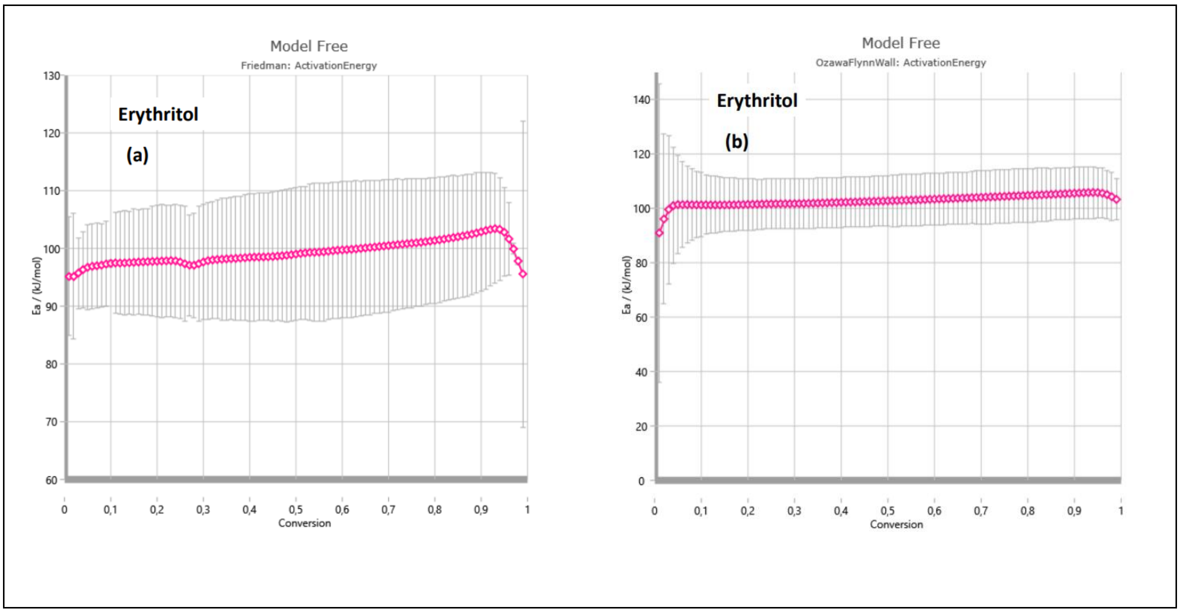

Figure S4. Graphic of E_a versus converted fraction (α) in (**a**) Friedman and (**b**) Ozawa-Fynn-Wall analysis for erythritol.

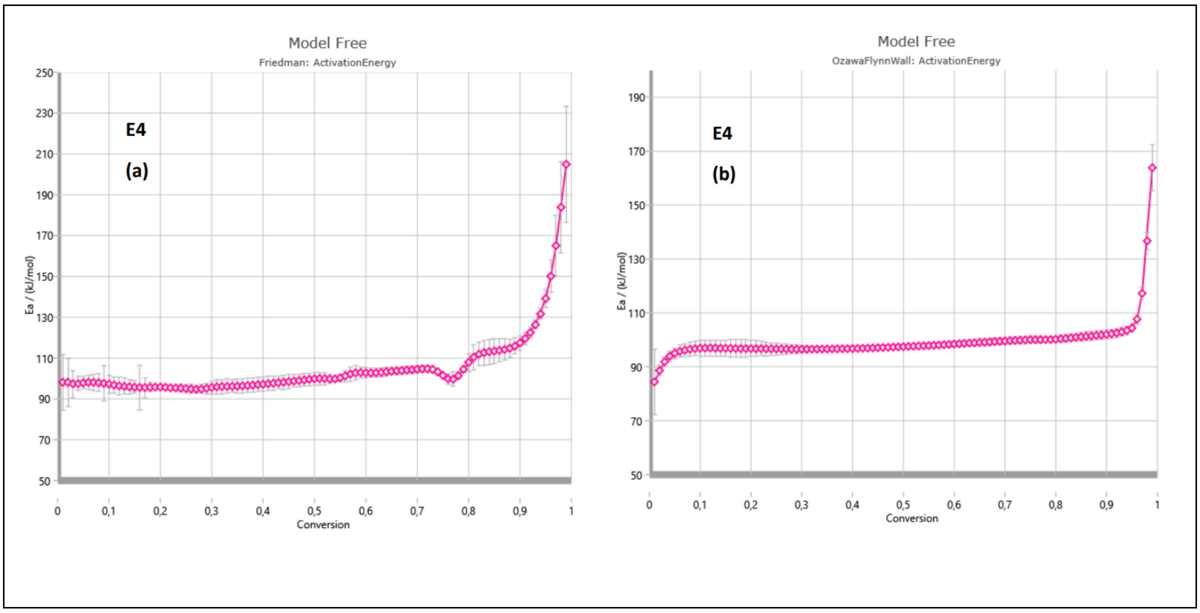

Figure S5. Graphic of E_a versus converted fraction (α) in (**a**) Friedman and (**b**) Ozawa-Fynn-Wall analysis for E4 sample.

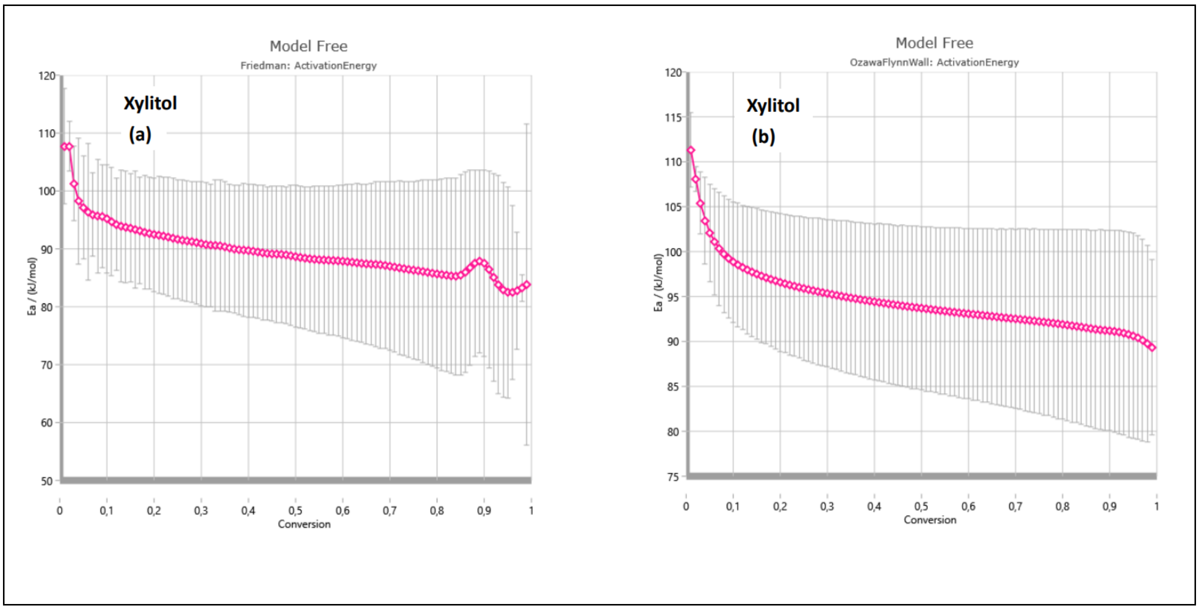

Figure S6. Graphic of E_a versus converted fraction (α) in (**a**) Friedman and (**b**) Ozawa-Fynn-Wall analysis for xylitol.
